# UNMET SERVICE NEEDS AMONG RURAL APPALACHIAN CAREGIVERS OF PEOPLE WITH ALZHEIMER’S AND DEMENTIA

**DOI:** 10.1093/geroni/igad104.1708

**Published:** 2023-12-21

**Authors:** David Russell, Christina Miyawaki, Erin Bouldin

**Affiliations:** Appalachian State University, Boone, North Carolina, United States; University of Houston, Houston, Texas, United States; University of Utah, Salt Lake City, Utah, United States

## Abstract

Risk factors for Alzheimer’s disease and related dementias (ADRD) are higher in rural areas than urban areas, and home- and community-based services (services) are less accessible. This study sought to identify unmet services needs among people with ADRD and their caregivers living in rural Appalachia, and highlight contextual factors that shape service access and utilization. We interviewed 22 caregivers and persons with ADRD living in Western North Carolina between August 2021 and April 2022 with recruitment assistance from local agencies and organizations. Themes were identified across domains of our conceptual model informed by theories of health services use, family caregiving, and sociocultural contexts. Family caregivers communicated multiple unmet needs, foremost of which included fragile and insufficient support systems or imbalances between family and paid caregiving support, a lack of clarity about their services options, and feeling behind in preparing for the future. In the presence of unmet care needs, family caregivers made decisions (or not) to use services in aiding them with caring for persons with ADRD. These decisions were shaped by illness-level (e.g. ADRD symptoms and progression), predisposing (e.g. work experiences), and enabling factors (e.g. family support). The sociocultural contexts in which family caregivers and the persons they cared for with ADRD were embedded within, including their culture, beliefs and family norms, also shaped perceptions and use of services. These contexts should be better understood and considered when developing and implementing acceptable services for people with ADRD in rural areas.

